# A novel MRI- and CT-based scoring system to differentiate malignant from osteoporotic vertebral fractures in Chinese patients

**DOI:** 10.1186/s12891-018-2331-0

**Published:** 2018-11-20

**Authors:** Zi Li, Ming Guan, Dong Sun, Yong Xu, Feng Li, Wei Xiong

**Affiliations:** 1 Department of orthopedics, Tongji Hospital, Tongji Medical College, Huazhong University of Science and Technology, 1095#, Jiefang Ave, Wuhan, Hubei China; 2 Department of orthopedics, Taikang Tongji Hospital, Wuhan, Hubei China; 3 Radiology department, Tongji Hospital, Tongji Medical College, Huazhong University of Science and Technology, 1095#, Jiefang Ave, Wuhan, Hubei China

**Keywords:** Computed tomography, Discriminant analysis, Magnetic resonance imaging, Malignant vertebral fracture, Osteoporotic vertebral fracture

## Abstract

**Background:**

Various types of magnetic resonance imaging (MRI) and computed tomography (CT) findings are used to differentiate malignant vertebral fractures (MVFs) from osteoporotic vertebral fractures (OVFs). The distinguishing ability of any single finding is limited. This study developed a novel scoring system that integrates multiple MRI and CT signs for improved accuracy of differential diagnosis between MVFs and OVFs.

**Methods:**

A total of 150 MVFs and 150 OVFs in thoracolumbar vertebrae were analyzed. MRI and CT images were obtained within 2 months of the probable time of fracture. The sensitivity and specificity of 15 MRI and CT image findings were evaluated. A stepwise discriminant analysis using these signs as variables was used to create a scoring system to differentiate MVFs from OVFs.

**Results:**

All 15 image findings had strong specificity and moderate sensitivity. Seven MRI and three CT image findings were selected and assigned integral values in the final scoring system. A total score of 4 or greater points indicated MVF, whereas a total score of 3 or fewer points indicated OVF. The classification accuracy was 98.3% in the test set.

**Conclusions:**

This novel scoring system using MRI and CT radiologic findings to differentiate MVFs from OVFs in Chinese patients was efficient with high accuracy and good applicability.

## Background

Vertebral fractures caused by benign or malignant lesions are common among the elderly. Identifying the etiology of spinal fractures at an early stage is critical to determine the clinical course, treatment, and prognosis [1–4]. Common features of osteoporotic vertebral fracture (OVF) and malignant vertebral fracture (MVF), including age group, clinical symptoms, and history of inadequate trauma, make differential diagnosis challenging. Open biopsy is considered the benchmark to diagnose musculoskeletal lesions, with 98% accuracy [5]. Its clinical application has been limited due to increased morbidity and a significant risk of complications [6, 7]. Percutaneous biopsy, a less invasive option recommended as an alternative for open biopsy, has a wide range of reported accuracy rates from 16 to 92% and a complication rate between 0 and 10% [8].

Modern radiological imaging techniques, including magnetic resonance imaging (MRI) and computed tomography (CT), have good predictive value for differential diagnosis. Multiple-image findings are utilized to distinguish between MVFs and OVFs [1, 3, 4, 9, 10]. Single-image findings have limited distinguishing ability and are not considered sufficiently sensitive or specific, such as fluid sign and pedicle involvement [1, 11, 12]. Misdiagnosis or delayed diagnosis of MVF is not uncommon in clinical practice, potentially due in part to confusing MRI and CT image findings. Unnecessary biopsy and pathological examinations of OVF patients diminishes the medical treatment experience and increases the risk and cost to the patient.

Integrating characteristic image findings can improve the accuracy of differential diagnosis. Discriminant analysis is a generally accepted statistical method that combines multiple features to separate or characterize two or more classes of clinical issues [13–15]. Two earlier studies attempted to create a scoring system to distinguish MVFs from OVFs using discriminant analysis, but the etiology types for malignant cases [16] or the sample sizes were limited [17].

This study utilized a large sample of Chinese patients to generate a novel scoring system to differentiate MVFs from OVFs using MRI and CT image findings.

## Methods

All OVF and MVF cases contained in the electronic records archives of the department of spine surgery for the period January 2013 to March 2018 were included in the study. Our institutional Ethics Review Board approved the study protocol. Informed and written concent was obtained from all patients. MRI images were obtained using two 3.0 T magnetic resonance scanners (Siemens Healthcare, Skyra, Germany; GE Healthcare, Discovery MR750, USA). Images of 5-mm thick contiguous or vertebral bodies and disc level CT sections were performed using a 16-detector CT scanner (GE Healthcare, LightSpeed 16, USA) and 1.25-3 mm thick reconstruction slices with no overlap were obtained for evaluation. The inclusion and exclusion criteria listed below were used to further refine selection of the sample.

### Inclusion criteria

1. A definitive diagnosis was required for inclusion. Patients included in the group of MVF required an exact pathologic diagnosis for one vertebrae (positive percutaneous transpedicular biopsy or pathological specimen through spinal surgery). OVF diagnosis was verified by benign histologic pathology, or not aggravated or improving clinical symptoms with restoration of vertebral signal intensity on MRI observed for a period of at least 2 months [16].
2. Only thoracolumbar vertebral lesions were included.
3. An MRI or CT was obtained within 2 months of the probable day of fracture.

### Exclusion criteria

1. Neurogenic tumor cases, such as schwannoma or neurofibroma.
2. MVF patients who had already received an operation, irradiation, or biopsy.
3. Concomitant cases of OVF and MVF.
4. Severe trauma cases, such as a traffic accident or high falling injury.

A total of 150 OVFs and 150 MVFs in 226 Chinese patients were selected. The 300 vertebral fracture images were reviewed by two orthopedic surgeons (Zi Li and Ming Guan each with 2 years of experience) and one musculoskeletal radiologist (Dong Sun with 2 years of experience). A total of 15 key findings (12 MRI and 3 CT) previously proposed in the literature was applied to image evaluation. Discrepancies were resolved by debate until consensus was achieved. The sensitivity and specificity of each finding was then calculated.

### MRI finding

#### Pattern change of vertebrae signal intensity

**Fig. 1:**
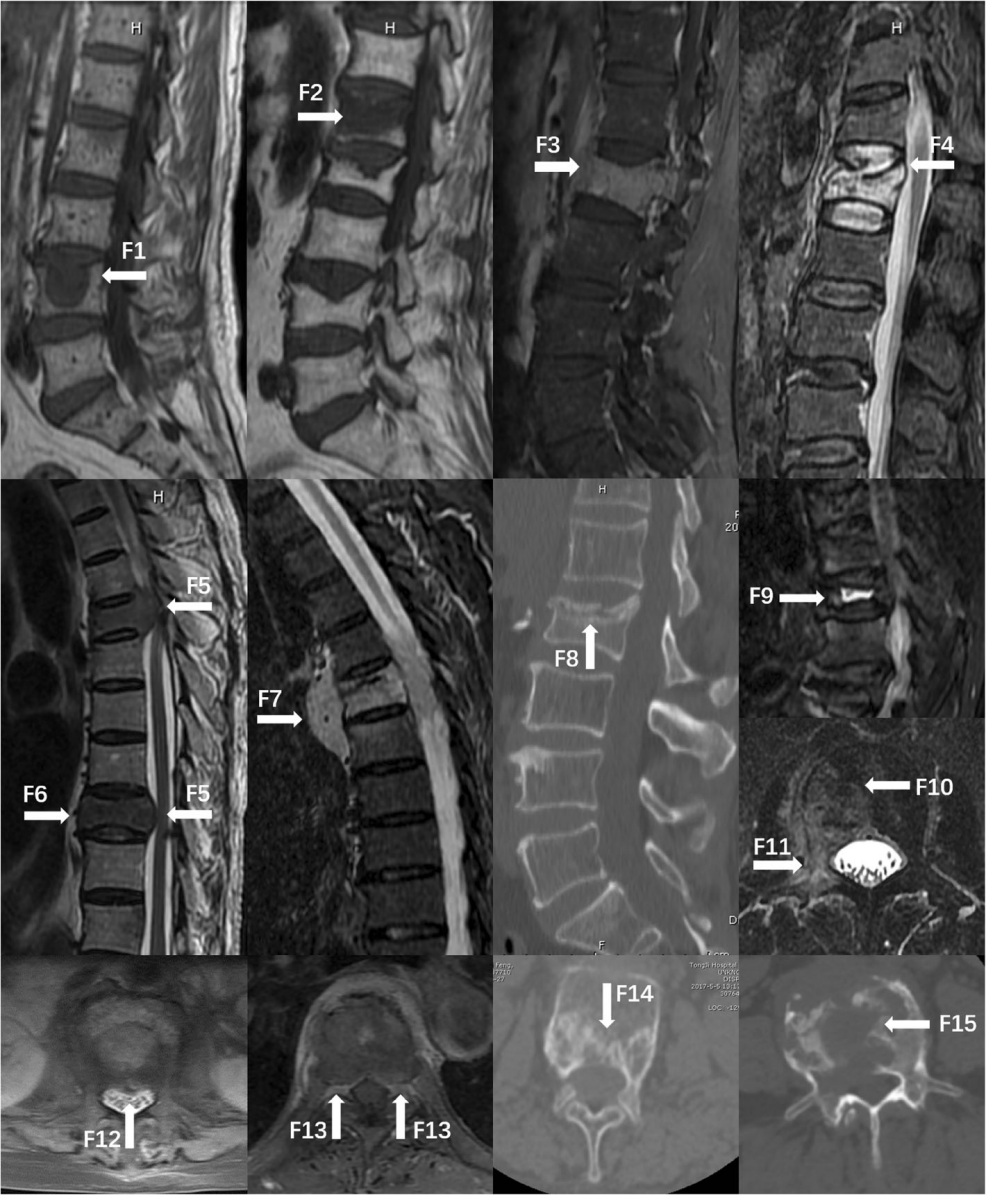
Key radiological magnetic resonance imaging (MRI) and computed tomography (CT) findings. F1 indicates a round vertebral signal intensity change (metastasis of prostate cancer); F2 indicates a band-like vertebral signal intensity change (osteoporotic vertebral fracture [OVF]); F3 indicates a diffuse vertebral signal intensity change (metastasis of lung cancer); F4 indicates a superior sharp protrusion of the posterior wall border (OVF); F5 indicates a smoothly blunt protrusion of the posterior wall border (metastasis of bladder cancer); F6 indicates an anterior vertebral convexity (metastasis of bladder cancer); F7 indicates a paravertebral solid mass (lymphoma); F8 indicates a sclerotic band beneath the end plate (OVF); F9 indicates a cleft fluid sign (OVF); F10 indicates an asymmetry in signal intensity (metastasis of kidney cancer); F11 indicates a pedicle involvement (metastasis of kidney cancer); F12 indicates a single-peaked posterior wall protrusion (OVF); F13 indicates a double-peaked posterior wall protrusion (metastasis of prostate cancer); F14 indicates a vertebral fracture without osteolysis (OVF); F15 indicates an osteolytic destruction (metastasis of lung cancer)

A lesion is likely malignant when the observed geometric pattern of vertebral signal intensity is round (Fig. 1, F1) [2, 12, 18, 19]. A band-like appearance is often seen in OVF (Fig. 1, F2) [12, 19, 20]. A whole vertebrae diffused with abnormal signal indicates MVF (Fig. 1, F3); OVF is implied if a normal signal remains [11, 12, 19, 21].

#### Contour of the anterior or posterior wall border

OVF is implied when there is a sharp protrusion in the posterior superior border of the vertebral body (Fig. 1, F4); a smoothly blunt protrusion in the posterior border of the vertebral body is often seen in MVF (Fig. 1, F5) [20, 22]. An anterior vertebral convexity indicates a higher likelihood of MVF than OVF (Fig. 1, F6) [23].

### Paravertebral solid mass

A paravertebral solid mass is more commonly detected in MVF than OVF (Fig. 1, F7). Spinal tumors are usually solid masses rather than cystic masses which are commonly detected in infectious cases, especially in the spinal tuberculosis [11, 18, 24].

### Cleft formation (“fluid sign”)

Fluid sign refers to a cleft configuration with a signal as low as that of water in T1WI and as high as that of water in T2WI (Fig. 1, F9) [2, 12, 19, 23, 24]. It supports a diagnosis of OVF rather than MVF.

### Asymmetry of signal intensity change in axial image

The symmetry of the signal intensity change in vertebrae indicates OVF, whereas asymmetry of the signal intensity change indicates MVF (Fig. 1, F10) [18].

### Pedicle involvement

A signal intensity change encompassing half of the pedicle is judged as pedicle involvement and indicates MVF (Fig. 1, F11) [12, 19, 20].

### Pattern of posterior wall protrusion in axial image

The normal pattern of posterior wall protrusion in OVF is single-peaked (Fig. 1, F12). The pattern of posterior wall protrusion in MVF is usually double-peaked (Fig. 1, F13) [18].

### CT imaging finding

#### Sclerotic band beneath the end plate

The sclerotic band refers to a zone of trabeculae compaction and late reactive callus that has a high density in CT after osteoporotic fractures, usually accompanied by a deformed end plate (Fig. 1, F8) [19, 25].

#### Relationship between fracture and osteolysis

An apparent vertebral fracture line without osteolysis, with no tumor cells invading, is often seen in OVF (Fig. 1, F14). The osteolytic destruction of the vertebral cortex or body is observed in 97% of MVF cases (Fig. 1, F15) [18, 19, 25].

After image evaluation, a stepwise discriminant analysis was used to produce the scoring system. Discriminant analysis is a generalized statistical method that generates a suitable combination of features that separate or characterize two or more classes of objects [26]. Each image finding was considered a dummy variable, and multiple linear regression analysis was implemented with corresponding discriminant coefficients as scores, and constants as discriminant threshold. The stepwise method selects the “best” variables automatically in the analysis when comparing many variables. Starting with a situation that does not include any variables, the variable with the largest *F to Enter* value greater than the entry criteria (set by default as 3.84) is included in the analysis at each step. The variables with *F to Enter* values < 3.84 are left out of the analysis until no more are added. The error rate of discrimination is calculated by the leave-one-out cross validation method: any sample is regarded as a test case and the remaining samples are regarded as a training set, detecting on the cycle until all samples have become test cases once. A novel scoring formula was obtained, with corresponding coefficients of selected predictors as scores and the discriminant threshold as a constant. All data were statistically analyzed using IBM SPSS version 19. Single parameters were analyzed using chi-square tests. A value of *p* < 0.05 was considered statistically significant.

## Results

The MVF group had a younger mean age and lower proportion of females than the OVF group. The most common metastasis and primary neoplasms were lung cancer (35 cases) and multiple myeloma (21 cases), respectively. The clinical characteristics of the included cases are presented in Table 1. The calculated sensitivity and specificity of the 15 image findings included in the analysis are presented in Table 2.

**Table 1:** Clinical characteristics

Characteristics	MVF	OVF
n (vertebrae)	150	150
n (patients)	106	126
Gender (Male/Female)	74/32	34/92
Age (years), Mean ± SD (range)	55.5 ± 11.9 (23–75)	66.3 ± 7.6 (51–82)
Spinal level		
Thoracic, n (%)	70 (46.7%)	69 (46.0%)
Lumbar, n (%)	80 (53.3%)	81 (54.0%)
n (tumor type)		N/A
Lung	35	
Multiple myeloma	21	
Prostate	13	
Bladder	11	
Liver	9	
Kidney	8	
Lymphoma	7	
Breast	4	
Leukemia	4	
Osteosarcoma	3	
PNET	3	
Chondrosarcoma	2	
Other	30	

*MVF* metastatic vertebral fracture, *OVF* osteoporotic vertebral fracture, *PNET* peripheral neuroectodermal tumor, *N/A* not applicable

**Table 2:** Sensitivity and specificity of key MRI and CT findings

Radiological Findings	Implication	Sensitivity (%)	Specificity (%)	*p*-value
MRI findings				
Pattern change of vertebrae signal intensity				
Round	MVF	43	99	< 0.001
Band like	OVF	58	97	< 0.001
Diffuse	MVF	79	91	< 0.001
Shape of posterior wall protrusion				
Superior sharp	OVF	43	87	< 0.001
Smoothly blunt	MVF	57	96	< 0.001
Anterior vertebral convexity	MVF	23	97	< 0.001
Paravertebral solid mass	MVF	63	99	< 0.001
Fluid sign	OVF	29	99	< 0.001
Asymmetry of signal intensity change	MVF	80	87	< 0.001
Pedicle involvement	MVF	86	79	< 0.001
Shape of posterior wall protrusion				
Single peaked	OVF	26	88	< 0.05
Double peaked	MVF	35	97	< 0.001
CT findings				
Sclerotic band beneath the end plate	OVF	65	99	< 0.001
Fracture without osteolysis	OVF	59	99	< 0.001
Osteolytic destruction	MVF	63	98	< 0.001

*CT* computed tomography, *MRI* magnetic resonance imaging, *MVF* malignant vertebral fracture, *OVF* osteoporotic vertebral fracture

### Discriminant analysis

The 15 image findings listed in Table 2 were set as independent variables. A variable present in one vertebra was set as 1; if it was not present, the value was set as 0. Stepwise discriminant analysis was then performed. A total of ten selected findings with the corresponding coefficients and the constant in the result are presented in Table 3; the remaining five findings were excluded because of low tolerance. The classification accuracy of the stepwise discriminant analysis was 98.3%, Wilks’ λ was 0.158 (*p* < 0.001), and the discriminant accuracy of the cross-validation (leave-one-out method) was 97.7%.

**Table 3:** Result of the discriminant analysis

Radiological Findings	Discriminant coefficient
MRI findings	
Pattern change of vertebrae signal intensity	
Round	3.794
Band like	−1.730
Diffuse	4.238
Smoothly blunt border protrusion of the posterior wall	1.909
Paravertebral solid mass	4.250
Asymmetry of signal intensity change	4.559
Pedicle involvement	4.330
CT findings	
Sclerotic band beneath the end plate	−3.467
Fracture without osteolysis	−3.786
Osteolytic destruction	3.051
Discriminant threshold	7.272

*CT* computed tomography, *MRI* magnetic resonance imaging, *MVF* malignant vertebral fracture, *OVF* osteoporotic vertebral fracture

Decimal scores are not practical in clinical settings. To enhance practicability, we divided the constant and coefficients term by two and the results were rounded into integral numbers. Finally, a simplified scoring system was created (Table 4). A total score ≥ 4 indicates MVF, whereas a total score ≤ 3 points indicates OVF. The classification accuracy of the simplified scoring system was 98.3%, equal to the original discriminant analysis. Only three OVF and two MVF cases were incorrectly classified by the simplified scoring system.

**Table 4:** Modified scoring system for diagnosis of malignant vertebral fractures (MVFs)

Radiological Findings	Implication	Score
MRI findings		
Pattern change of vertebrae signal intensity		
Round	MVF	2
Band-like	OVF	−1
Diffuse	MVF	2
Smoothly blunt border protrusion of the posterior wall	MVF	1
Paravertebral solid mass	MVF	2
Asymmetry of signal intensity change	MVF	2
Pedicle involvement	MVF	2
CT findings		
Sclerotic band beneath the end plate	OVF	−2
Fracture without osteolysis	OVF	−2
Osteolytic destruction	MVF	2
Total score: OVF ≤ 3 and MVF ≥ 4		

*CT* computed tomography, *MRI* magnetic resonance imaging, *MVF* malignant vertebral fracture, *OVF* osteoporotic vertebral fracture

## Discussion

A stepwise discriminant analysis was used to create a novel simple scoring system to differentiate MVF from OVF using MRI and CT image findings, with a discriminant accuracy of 98.3%.

Relatively low sensitivity and high specificity were calculated for image findings. The distinguishing ability of any single radiological finding was limited. The rational application of discriminant analysis to integrate different image findings was a statistically valid and logical method to maximize the diagnostic accuracy rate. A total of 15 significant image findings were included as variables in the study based on previous research. Analysis of the input variable contribution to the output eliminated five findings, and the rational model was established. Only two previous studies have attempted to develop scoring systems to differentiate benign and malignant vertebral fractures using discriminant analysis [16, 17]. The score scale of the study from Yuzawa et al. was also based on MRI and CT signs, but had a limited sample size (100 cases), and radiologic findings yielded relatively low accuracy rates [17]. This study included four involved findings (pedicle involvement, paravertebral solid mass, fracture without osteolysis, and osteolytic destruction) not included in previous scoring systems.

The discriminant accuracy based on MRI or CT findings alone were 96.0 and 89.7%, respectively. These are slightly lower than the MRI findings accuracy of 96.6% reported by So Koto et al., who concluded that using MRI findings alone provides satisfactory accuracy for differential diagnosis [16]. Including findings, image judging criteria, sample size, and ethnicity may account for the different accuracy rates. To improve accuracy, this study included CT image findings in the analysis. CT is the most suitable imaging technique to identify calcification, ossification, extent of lesion, and cortical outline with high spatial resolution [24]. The affordability and speed of CT scanning have made it a general imaging service in China. Supplementing MRI findings with CT are indispensable for differential diagnosis.

The sclerotic band beneath the end plate was an important finding in the scoring system. A faint band of sclerosis beneath the end plate refers to a zone of trabeculae compaction and late reactive callus, which has high density in CT after osteoporotic fracture and may be present for 8 to 10 weeks post-fracture [19]. Reactive sclerotic change was observed with 77.8% sensitivity and 90.0% specificity, indicating that sclerotic change on CT images was a statistically significant finding indicating benign lesion [25]. The discriminant accuracy decreased to 97.3% without the sclerotic band beneath the end plate, verifying the importance of the finding.

A total of 300 cases were included in this study, larger than any other sample in the literature. The tumor etiology was primarily primary and metastatic tumors. Pathological examination is considered the benchmark for tumor diagnosis, and all patients of MVF included in this study had exact pathologic diagnoses of one vertebrae (positive percutaneous transpedicular biopsy or pathological specimen through spinal surgery). In the study from Kato et al., metastatic tumor cases were diagnosed either by pathologic diagnosis or malignancy radiographic changes, which are less rigorous than the criteria used in this study [16].

Combining 15 MRI and CT findings based on the available literature, and selecting 10 variables for the scoring system, yielded a system with discriminant accuracy higher than that achieved in previous studies.

There are several limitations to this study. Firstly, although CT findings can improve the discriminant accuracy for differentiating MVFs from OVFs, the hazards of radiation ionization would not be ignored. Secondly, there were many multiple malignant vertebral fractures in the available cases. Single vertebral involvement is the best operation indication for *en bloc* resection in malignant vertebral fractures [27]. One of the original objectives for this study was to contribute to the surgical decision for single vertebral pathologic fractures. Due to the limited sample size, vertebrae were used as the sample unit for assessment rather than patients. A multi-center approach to compile more cases of single pathological vertebral fracture can be used address this issue in the future. Additionally, clinical information including symptoms and tumor markers can assist clinicians with diagnoses and may be added to a future scoring system.

## Conclusion

A novel scoring system based on MRI and CT image findings in Chinese patients was effective and convenient for differentiating MVFs from OVFs. A future multi-center validation is required to confirm the accuracy and practicability of the scoring system.

## Abbreviations

CT: Computed tomography
MRI: Magnetic resonance imaging
MVF: Malignant vertebral fracture
OVF: Osteoporotic vertebral fracture
